# A Novel Approach to Recurrent Hepatic Hydatid Cyst Using EUS‐Guided Aspiration and Ethanol Injection: A Case Report and Focused Literature Review

**DOI:** 10.1155/crgm/5497218

**Published:** 2026-04-28

**Authors:** Pouyan Ebrahimi, Amin Esmaeilnia Shirvani, Mohammad Golparvar Azizi, Hadie Razjouyan, Seyed Hassan Abedi

**Affiliations:** ^1^ School of Medicine, Babol University of Medical Sciences, Babol, Iran, mubabol.ac.ir; ^2^ Department of Internal Medicine, Rouhani Hospital, Babol University of Medical Sciences, Babol, Iran, mubabol.ac.ir; ^3^ Associate Professor of Medicine, Interventional Gastroenterologist, Milton S. Hershey Medical Center, Penn State University, Hershey, 17033, Pennsylvania, USA, psu.edu; ^4^ Assistant Professor of Gastroenterology and Hepatology, Department of Internal Medicine, School of Medicine, Babol University of Medical Sciences, Babol, Iran, mubabol.ac.ir

**Keywords:** *Echinococcus*, endoscopy, EUS, hydatid cyst, liver

## Abstract

**Background:**

Hepatic hydatid disease, a manifestation of cystic echinococcosis caused by *Echinococcus granulosus*, remains endemic in many regions and poses a persistent therapeutic challenge. Although surgery is often employed for definitive management, recurrence, procedural risks, and anatomical constraints have led to growing interest in minimally invasive alternatives such as percutaneous aspiration and endoscopic interventions.

**Case Presentation:**

We report a 52‐year‐old male with an incidentally detected hepatic hydatid cyst who initially underwent surgical cystectomy and cholecystectomy. Twenty‐five days postoperatively, the patient developed new abdominal fullness, and repeat imaging revealed a cystic lesion in the left hepatic lobe measuring 13.4 × 9.9 cm, reported radiologically as recurrence. The patient underwent endoscopic ultrasound (EUS)–guided fine‐needle aspiration and lavage of the cyst cavity with 20 mL of 96% ethanol. The procedure was well tolerated with no adverse events, and follow‐up imaging at 6 months confirmed complete cyst resolution.

**Conclusion:**

This case demonstrates the technical feasibility and clinical safety of EUS‐guided ethanol ablation as a minimally invasive alternative for recurrent hepatic hydatid cysts. It highlights the expanding therapeutic potential of EUS beyond conventional indications and supports further exploration of this technique in selected cases.

## 1. Introduction

Cystic echinococcosis, commonly known as hydatid disease, is a parasitic zoonosis caused by the larval stage of *Echinococcus granulosus*, with the liver being the most frequently affected organ in humans [1]. The disease remains endemic in several regions, including the Middle East, North Africa, South America, and parts of Asia. Clinical presentation varies from asymptomatic incidental findings to complications such as cyst rupture, biliary obstruction, or anaphylactic shock [2]. Given its potential for serious complications, hydatid liver disease has prompted the development of multiple therapeutic strategies over time. For decades, surgery, either conservative or radical, has been the cornerstone of treatment, particularly for large, symptomatic, or complicated cysts. However, surgical approaches are often associated with significant morbidity, recurrence, and prolonged hospitalization [3]. Pharmacotherapy using benzimidazole derivatives such as albendazole is another mainstay, particularly in inoperable cases or for perioperative prophylaxis, though its efficacy remains limited when used alone, with a complete response rate ranging from only 20% to 30% [4]. In the past 2 decades, image‐guided percutaneous techniques, especially the puncture–aspiration–injection–reaspiration (PAIR) method, have emerged as a minimally invasive and effective alternative for selected patients, particularly for Type I and II cysts as per WHO classification [5]. The use of scolicidal agents such as hypertonic saline or absolute alcohol has demonstrated high efficacy and low recurrence rates when performed under ultrasonographic or CT guidance [6].

Despite the advances in image‐guided therapy, the application of endoscopic ultrasound (EUS) as a guiding modality for aspiration and injection in hepatic hydatid cysts remains undocumented in the literature. EUS has proven utility in accessing intra‐abdominal lesions adjacent to the gastrointestinal tract with high precision and minimal invasiveness [7], yet its role in parasitic cyst management is rarely explored [8]. In this report (that evaluated with the CARE checklist [Appendix 1]), we present an adult case of recurrent hepatic hydatid disease successfully managed with EUS‐guided aspiration and ethanol ablation, marking what may be the first documented use of this minimally invasive technique in such pathology.

## 2. Case Presentation

**FIGURE 1:**
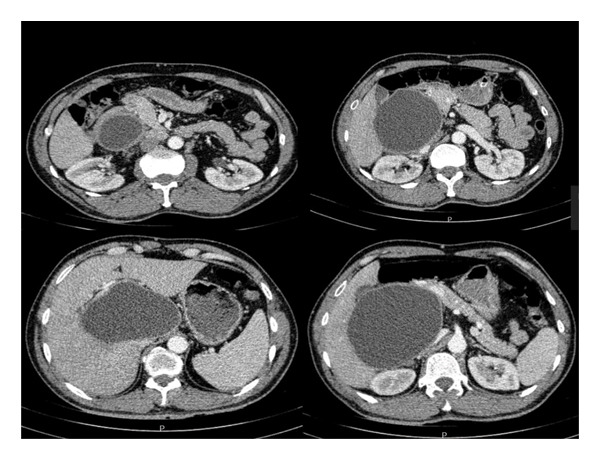
Axial contrast‐enhanced CT images of the abdomen demonstrating a large, well‐circumscribed, unilocular hydatid cyst in the right hepatic lobe. The cystic lesion measures approximately 135 × 103 mm and exhibits a sharply defined wall with homogeneous fluid attenuation, consistent with a CE1‐type cyst according to WHO classification. The lesion exerts a significant extrinsic mass effect on adjacent vascular structures, notably the portal vein and inferior vena cava (IVC), without evidence of intralesional calcification or biliary ductal dilation. No septations, daughter cysts, or signs of intrabiliary rupture are visualized, supporting the diagnosis of a primary unilocular cystic echinococcal lesion.

A 52‐year‐old Caucasian male was incidentally found to have a hepatic cystic lesion during evaluation for a motor vehicle accident in December 2019 at Rouhani Hospital, Babol, Iran. The patient was entirely asymptomatic, with no abdominal discomfort, systemic symptoms, or notable medical or familial history. He denied smoking, alcohol use, and recreational drug consumption and reported no known allergies. On physical examination, his vital signs were stable: blood pressure 120/70 mmHg, heart rate 75 bpm, respiratory rate 16 breaths per minute, and axillary temperature 36.0°C. Both systemic and neurological examinations were unremarkable. Laboratory studies, including complete blood count and liver and renal function tests, were within normal limits. Initial ultrasonography of the abdomen revealed a large cystic lesion, prompting further evaluation with contrast‐enhanced computed tomography scan (CT scan). Imaging demonstrated a well‐circumscribed, unilocular cystic lesion measuring approximately 14 × 10 cm in the hepato‐duodenal ligament and porta hepatis, exerting mass effect on the portal vein and adjacent duodenum, without biliary tract dilatation or intralesional septations (Figure 1). Multiple small calcified gallstones were noted in the gallbladder.

**FIGURE 2:**
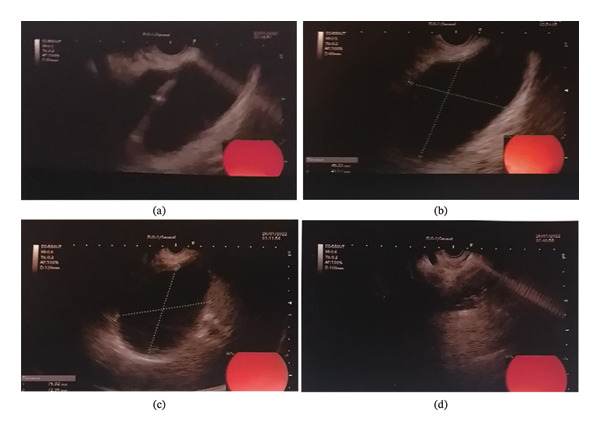
Multimodal ultrasonographic evaluation of a recurrent hepatic hydatid cyst before and after EUS‐guided intervention. Transabdominal ultrasound images demonstrate a large, anechoic, unilocular cystic lesion in the left hepatic lobe, measuring 134 × 99 mm, consistent with a WHO CE1 hydatid cyst. The cyst exhibits a well‐defined margin with posterior acoustic enhancement and no internal septations or daughter cysts (a and b). Linear‐array EUS image showing the same lesion with a slightly smaller measured diameter of 75 × 74 mm due to transgastric proximity and differing imaging planes. The cyst appears anechoic with a thin, regular wall and no evidence of biliary communication (c). Postprocedural EUS image obtained following complete cyst aspiration and intralesional instillation of 20 mL of 96% ethanol, showing near‐collapse of the cyst cavity and the presence of fine internal echoes, likely representing fluid–alcohol interface or initial coagulative changes (d).

Given the cyst’s size and compressive features, an infectious disease specialist was consulted as part of the multidisciplinary evaluation. Although imaging findings were highly suggestive of hydatid disease, empirical intravenous antibiotic therapy (ciprofloxacin 400 mg every 12 h and metronidazole 500 mg every 8 h for 3 days) was initiated based on specialist recommendation to cover the possibility of secondary infection. Based on typical imaging characteristics, a presumptive diagnosis of a hepatic hydatid cyst was made. Despite the lack of serological confirmation, the lesion’s size and compressive features warranted surgical intervention. The patient subsequently underwent open surgical resection of the cyst along with cholecystectomy. Histopathological examination confirmed *E. granulosus* infection. However, 25 days after surgery, the patient developed a sensation of fullness in the left upper abdominal region. Given the new onset of symptoms, a repeat abdominal ultrasound was performed, which revealed a newly detected cystic lesion, now measuring 13.4 × 9.9 cm, this time located in the left hepatic lobe. The radiology report described this finding as recurrence; however, the possibility of a residual or incompletely drained cyst could not be entirely excluded. A multidisciplinary team review favored a minimally invasive approach over reoperation. The patient consented to undergo EUS‐guided intervention, and the procedure was scheduled accordingly. Preintervention imaging confirmed the presence of a unilocular cyst without septation or biliary communication (Figures 2(a), 2(b), and 2(c)).

During the procedure, the patient received prophylactic intravenous antibiotics and was sedated. A linear‐array echoendoscope was advanced to visualize the cyst from the gastric lumen. A 19‐gauge Acquire needle (Boston Scientific) was inserted into the lesion under EUS guidance, and clear, nonbilious fluid was aspirated to dryness. Following complete aspiration, 20 mL of 96% absolute alcohol was instilled into the cavity and retained to achieve scolicidal activity (Figure 2(d)). No immediate or delayed complications were observed. The patient was monitored and discharged the next day in stable condition. At 6‐month follow‐up, repeat imaging confirmed complete resolution of the cystic lesion with no evidence of recurrence. The patient remained asymptomatic and in good general health.

## 3. Discussion

Surgical intervention has long been considered the gold standard in the treatment of hepatic hydatid cysts, particularly for large, symptomatic, or complicated lesions. Traditional approaches include radical resections (such as hepatectomy or pericystectomy) and more conservative procedures like partial cystectomy or deroofing. However, accumulating evidence over the past 2 decades has challenged the exclusive role of surgery. In a 20‐year retrospective study by Mahmoodi et al., postoperative recurrence occurred in 8.2% of patients, the majority of whom had not received adjunctive albendazole therapy, highlighting both the limitations and risks associated with surgery alone [9]. Likewise, Touati et al. emphasized that although conservative surgery can be successful, it remains prone to complications such as biliary fistulas and intra‐abdominal infections, particularly in giant or multivesicular cysts [10]. These findings support the growing consensus that while surgery retains a critical role, particularly for complicated cysts or cases with biliary communication, its drawbacks, including morbidity, technical demands, and resource intensity, necessitate alternative therapeutic strategies.

The introduction of antiparasitic therapy with benzimidazoles, particularly albendazole, has significantly impacted hydatid disease management. Albendazole is used both preoperatively to reduce cyst viability and postoperatively to decrease recurrence risk. While monotherapy with albendazole may induce cyst degeneration in select cases, its overall curative efficacy remains modest. A landmark observational study by Horton et al. found that only 28.5% of patients treated with albendazole alone achieved complete cure, while over 50% showed a partial response [11]. More recent long‐term retrospective analyses reaffirm that albendazole alone is rarely sufficient for large or complex cysts, although its perioperative use significantly reduces recurrence and operative complexity [9, 12].

The emergence of percutaneous techniques, particularly the PAIR method, marked a paradigm shift in the nonsurgical treatment of hepatic echinococcosis. First described in the 1980s, PAIR has been endorsed by the WHO for selected CE1 and CE3a cysts, especially in settings with expertise in interventional radiology. Studies have demonstrated that PAIR, when combined with albendazole, yields comparable or even superior efficacy to surgery in terms of cyst resolution, with significantly reduced hospital stay and complication rates [13]. In a prospective randomized trial, PAIR achieved cyst disappearance in 88% of cases, compared to 72% in the surgical arm, with notably fewer complications. More recently, advanced modifications of PAIR, including catheter drainage and the Örmeci technique, have expanded indications to more complex cysts, although these methods are still limited by cyst location, operator expertise, and risk of anaphylaxis or secondary infection [14]. Despite the availability of these established modalities, hepatic hydatid disease remains challenging in certain clinical scenarios, particularly in cases of recurrence, deep‐seated cysts, or poor surgical candidacy, thus warranting continued exploration of novel, less invasive treatment pathways.

Advancements in diagnostic imaging have fundamentally reshaped the therapeutic landscape of hepatic echinococcosis. While abdominal ultrasonography remains the cornerstone for the initial diagnosis and WHO classification of cysts, cross‐sectional modalities such as CT scan and magnetic resonance imaging (MRI) provide enhanced anatomic delineation, especially valuable in preoperative planning and detecting biliary communication or daughter cysts. These imaging tools have also enabled the evolution of minimally invasive interventions, with ultrasound‐ or CT‐guided PAIR therapy emerging as a validated, cost‐effective, and low‐risk treatment modality for selected cyst types [6, 15]. Despite the efficacy of these external approaches, limitations persist, particularly in deep‐seated or posteriorly located hepatic cysts that are challenging to access percutaneously.

EUS, introduced initially as a diagnostic tool for pancreaticobiliary disorders, has evolved into a powerful interventional modality. With its ability to provide high‐resolution, real‐time visualization of structures adjacent to the gastrointestinal tract, EUS permits precise access to intra‐abdominal lesions that would otherwise require surgical exposure. Its utility in aspiration and ablation of pancreatic cysts, neuroendocrine tumors, and even hepatic metastases is now well documented [16, 17]. In the context of hepatic hydatid cysts, however, the application of EUS has been exceptionally rare. Most literature to date has focused on percutaneous routes, with only a handful of reports exploring EUS‐guided therapy. For example, Barresi et al. described successful ethanol ablation of pancreatic remnants using EUS in postsurgical patients, noting good local control with minimal complications [18]. Similarly, Ferreira et al. reported regression of a large intra‐abdominal hemangioma using serial EUS‐guided ethanol injections when surgery was not feasible [19]. These findings suggest a broader potential role for EUS beyond its traditional domains.

Unlike percutaneous imaging, which may be limited by bowel interposition or difficult anatomic windows, EUS can provide transgastric or transduodenal access to hepatic segments II, III, IV, and parts of the caudate lobe with high fidelity [20]. Moreover, the use of linear echoendoscopes enables real‐time needle visualization, reducing the risk of vascular injury and facilitating complete aspiration of cyst content, essential for safe and effective instillation of scolicidal agents such as 96% ethanol [21, 22]. Although the application of EUS in hepatic hydatid disease is novel, several reports have documented its use in managing nonparasitic hepatic cysts. In a landmark study by Lee et al., ethanol lavage under EUS guidance was successfully performed in eight patients with large benign hepatic cysts, achieving near‐complete radiologic resolution at the 15‐month follow‐up [23]. Similarly, Lee and Seo expanded on this technique, treating 22 hepatic cysts via EUS‐guided ethanol retention, demonstrating excellent safety and efficacy, and establishing its utility for left‐lobe lesions with transgastric access [24]. Additionally, Hu et al. described a case of hepatic metastasis managed through EUS‐guided ethanol injection, reinforcing its role in accessing challenging hepatic sites adjacent to the gastrointestinal lumen [17]. Collectively, these studies confirm the feasibility of EUS‐guided aspiration and sclerotherapy in hepatic lesions, though none had addressed its application in parasitic cysts.

A recently reported case by Kumar and Sardiwalla highlighted the feasibility of EUS‐guided hepatic hydatid cyst management using a lumen‐apposing metal stent (LAMS) for transmural drainage [8]. Their approach involved deploying a Hot AXIOS stent to create a fistulous tract between the gastric lumen and the cyst cavity, facilitating continuous drainage and allowing for endoscopic debridement as needed. While this innovative method showed clinical success and cyst size reduction, it introduced potential risks such as stent migration, fistula persistence, and bleeding, complications reported in the broader literature on LAMS‐based drainage [25, 26]. In contrast, the patient in our report underwent a single‐session EUS‐guided fine‐needle aspiration of the recurrent hepatic hydatid cyst followed by intralesional injection of 20 mL of 96% ethanol, a scolicidal agent with long‐established efficacy. This approach eliminated the need for stent placement and continuous drainage, thereby minimizing the risk of delayed complications such as infection, bleeding, or stent dislodgment. Furthermore, the cyst was located in the left hepatic lobe and was directly accessible from the gastric wall via linear echoendoscopy, allowing real‐time visualization and safe needle guidance. No intra‐ or postprocedural adverse events occurred, and 6‐month follow‐up imaging confirmed complete cyst resolution without recurrence or residual symptoms. Importantly, our technique avoids reliance on costly hardware such as LAMS, rendering it more accessible and feasible in resource‐limited, hydatid‐endemic regions. While both EUS‐based approaches avoid repeat laparotomy, their methodological divergence is substantial. The Kumar case applies continuous stent‐mediated drainage, adapted from pancreatic fluid management, whereas our case employs direct scolicidal ablation under EUS guidance. Given the antigenic and infectious nature of hydatid fluid, avoiding a permanent fistulous tract may confer additional safety, though this hypothesis remains to be tested. In summary, our case highlights a streamlined, cost‐effective, and safe technique that broadens the therapeutic scope of EUS in hepatic echinococcosis.

## 4. Conclusion

This case demonstrates the feasibility and safety of EUS‐guided ethanol ablation as a novel, minimally invasive approach for treating recurrent hepatic hydatid cysts. Given its precision, cost‐effectiveness, and favorable short‐term outcome, this technique warrants further exploration. Given its promising outcome, further studies are warranted to evaluate its effectiveness compared to established approaches and to help inform future clinical protocols.

**Nomenclature**
ALT: Alanine aminotransferase
ALP: Alkaline phosphatase
AST: Aspartate aminotransferase
BUN: Blood urea nitrogen
BPM: Beats per minute
CE: Cystic echinococcosis
CT: Computed tomography
EUS: Endoscopic ultrasound
FNA: Fine‐needle aspiration
HCT: Hematocrit
HGB: Hemoglobin
LAMS: Lumen‐apposing metal stent
MCV: Mean corpuscular volume
MRI: Magnetic resonance imaging
PAIR: Puncture–aspiration–injection–reaspiration
RBCs: Red blood cells
WBCs: White blood cells

## Author Contributions

Conception and design: All authors. Administrative support: Pouyan Ebrahimi and Seyed Hassan Abedi. Provision of study materials or patients: Mohammad Golparvar Azizi, Pouyan Ebrahimi, and Amin Esmaeilnia Shirvani. Collection and assembly of data: Mohammad Golparvar Azizi and Seyed Hassan Abedi. Data analysis and interpretation: N/A. Manuscript writing: All authors. Final approval of manuscript: All authors.

## Funding

This research did not receive any specific grant from funding agencies in the public, commercial, or not‐for‐profit sectors.

## Ethics Statement

Ethical approval was not required for this case report in accordance with the policies of our institution. However, the case was formally registered at our institution with the tracking code 724136853. This study did not include experiments on animals or humans. The patients consented to the use of their data for this case report.

## Consent

Written informed consent was obtained from the patient for publication of this case report and any accompanying images. All personal identifying information has been anonymized.

## Conflicts of Interest

The authors declare no conflicts of interest.

## Supporting Information

Appendix. 1: CARE Checklist of information to include when writing a case report.

## Supporting information


**Supporting Information** Additional supporting information can be found online in the Supporting Information section.

## Data Availability

The data supporting the findings of this study are available upon request from the corresponding author and with permission from Babol University of Medical Sciences, Babol, Iran.
