# Supplementary material for: A Novel Approach to Recurrent Hepatic Hydatid Cyst Using EUS‐Guided Aspiration and Ethanol Injection: A Case Report and Focused Literature Review
Source: Case Rep Gastrointest Med. 2026 Apr 28;2026:5497218. doi: 10.1155/crgm/5497218 (PMC13122556; doi:10.1155/crgm/5497218)
Supplement: Supplementary file 1 — Supporting Information Additional supporting information can be found online in the Supporting Information section. [file CRGM-2026-5497218-s001.pdf]

CARE case report guidelines

Appendix.1: CARE Checklist of information to include when writing a case report

(cc) BY-NC-ND

Topic	Item	Checklist item description	Reported on Line
**Title**	**1**	The diagnosis or intervention of primary focus followed by the words "case report"	2
**Key Words**	**2**	2 to 5 key words that identify diagnoses or interventions in this case report, including "case report"	50
**Abstract (no references)**	**3a**	Introduction: What is unique about this case and what does it add to the scientific literature?	87-94
	**3b**	Main symptoms and/or important clinical findings	96-112
	**3c**	The main diagnoses, therapeutic interventions, and outcomes	113-133
	**3d**	Conclusion—What is the main "take-away" lesson(s) from this case?	125-133
**Introduction**	**4**	One or two paragraphs summarizing why this case is unique (**may include** references)	229-235
**Patient Information**	**5a**	De-identified patient specific information	Figures and lines 113-124
	**5b**	Primary concerns and symptoms of the patient	96-112
	**5c**	Medical, family, and psycho-social history including relevant genetic information	96-112
	**5d**	Relevant past interventions with outcomes	96-112
**Clinical Findings**	**6**	Describe significant physical examination (PE) and important clinical findings	96-112
**Timeline**	**7**	Historical and current information from this episode of care organized as a timeline	96-124
**Diagnostic Assessment**	**8a**	Diagnostic testing (such as PE, laboratory testing, imaging, surveys).	113-130
	**8b**	Diagnostic challenges (such as access to testing, financial, or cultural)	113-130
	**8c**	Diagnosis (including other diagnoses considered)	113-130
	**8d**	Prognosis (such as staging in oncology) where applicable	125-133
**Therapeutic Intervention**	**9a**	Types of therapeutic intervention (such as pharmacologic, surgical, preventive, self-care)	113-130
	**9b**	Administration of therapeutic intervention (such as dosage, strength, duration)	113-130
	**9c**	Changes in therapeutic intervention (with rationale)	113-117
**Follow-up and Outcomes**	**10a**	Clinician and patient-assessed outcomes (if available)	131-133
	**10b**	Important follow-up diagnostic and other test results	125-133
	**10c**	Intervention adherence and tolerability (How was this assessed?)	131-133
	**10d**	Adverse and unanticipated events	131-133
**Discussion**	**11a**	A scientific discussion of the strengths AND limitations associated with this case report	212-235
	**11b**	Discussion of the relevant medical literature **with references**	195-235
	**11c**	The scientific rationale for any conclusions (including assessment of possible causes)	237-241
	**11d**	The primary "take-away" lessons of this case report (without references) in a one paragraph conclusion	232-235
**Patient Perspective**	**12**	The patient should share their perspective in one to two paragraphs on the treatment(s) they received	133
**Informed Consent**	**13**	Did the patient give informed consent? Please provide if requested	**Yes** ✓ **No** ☐
